# Supplementary material for: Synaptic input sequence discrimination on behavioral timescales mediated by reaction-diffusion chemistry in dendrites
Source: eLife. 2017 Apr 19;6:e25827. doi: 10.7554/eLife.25827 (PMC5426902; doi:10.7554/eLife.25827)
Supplement: Source code 1. — The contents of the zip file include a README.txt, which has the file information and running instructions. In addition, it has script files for various parts of the figures, as well as model specification files for the chemical channel, and morphological parameters. Files include: DOI: http://dx.doi.org/10.7554/eLife.25827.019 [file elife-25827-code1.zip › Supplementary/TabulatedChemElecModels.docx]

**Supplementary Material**

Chemical model: Reduced MAPK system. All volumes are initial reference volumes, and are rescaled to actual volumes defined by detailed morphology of simulated neuron and subsequent spatial discretization for solving PDEs.

| PSD | Vol = 0.01 fl |  |  |
| --- | --- | --- | --- |
| Reactions | Kf | Kb |  |
| Ca_input <===> Ca | 500 s^-1 | 10 s^-1 |  |
| CaM-Ca3 + Ca <===> CaM-Ca4 | 1.8 uM^-1.s^-1 | 10 s^-1 |  |
| CaM + Ca <===> CaM-Ca | 8.4846 uM^-1.s^-1 | 8.4853 s^-1 |  |
| CaM-Ca2 + Ca <===> CaM-Ca3 | 3.6001 uM^-1.s^-1 | 10 s^-1 |  |
| CaM-Ca + Ca <===> CaM-Ca2 | 8.4846 uM^-1.s^-1 | 8.4853 s^-1 |  |
|  |  |  |  |
| Pools |  |  |  |
| name | Initial Concen | buffered | D (μm^2/s) |
| Ca | 0.1 uM | No | 100 |
| Ca_input | 0.08 uM | Yes | 0 |
| CaM | 40 uM | No | 0.5 |
| CaM-Ca3 | 0 uM | No | 1 |
| CaM-Ca2 | 0 uM | No | 1 |
| CaM-Ca | 0 uM | No | 1 |
| CaM-Ca4 | 0 uM | No | 1 |
|  |  |  |  |
| Spine Head | Vol = 0.09 fl |  |  |
| Reactions | Kf | Kb |  |
| CaM-Ca3 + Ca <===> CaM-Ca4 | 1.8 uM^-1.s^-1 | 10 s^-1 |  |
| CaM + Ca <===> CaM-Ca | 8.4845 uM^-1.s^-1 | 8.4853 s^-1 |  |
| CaM-Ca2 + Ca <===> CaM-Ca3 | 3.6001 uM^-1.s^-1 | 10 s^-1 |  |
| CaM-Ca + Ca <===> CaM-Ca2 | 8.4845 uM^-1.s^-1 | 8.4853 s^-1 |  |
| CaM <===> CaM_xchange | 1 s^-1 | 100 s^-1 |  |
|  |  |  |  |
| Pools |  |  |  |
| name | InitialConc | buffered | D (μm^2/s) |
| Ca | 0.11111 uM | No | 100 |
| CaM | 40 uM | No | 0.5 |
| CaM-Ca3 | 0 uM | No | 1 |
| CaM-Ca2 | 0 uM | No | 1 |
| CaM-Ca | 0 uM | No | 1 |
| CaM-Ca4 | 0 uM | No | 1 |
| CaM_xchange | 0 uM | No | 20 |
|  |  |  |  |
| Dendrite | Vol = 1 fl |  |  |
| Reactions | Kf | Kb |  |
| AA <===> APC | 0.4 s^-1 | 0.01 s^-1 |  |
| 2 Ca + Raf <===> act_Raf | 12 uM^-2.s^-1 | 4 s^-1 |  |
| K_A_p <===> K_A | 0.05 s^-1 | 0 s^-1 |  |
| 2 AA + PKC <===> act_PKC | 1 uM^-2.s^-1 | 2 s^-1 |  |
| Ca_input <===> Ca | 500 s^-1 | 10 s^-1 |  |
| reg_phosphatase <===> inact_phosphatase | 0.03 s^-1 | 0 s^-1 |  |
| CaM-Ca3 + Ca <===> CaM-Ca4 | 1.8 uM^-1.s^-1 | 10 s^-1 |  |
| CaM + Ca <===> CaM-Ca | 8.4846 uM^-1.s^-1 | 8.4853 s^-1 |  |
| CaM-Ca2 + Ca <===> CaM-Ca3 | 3.6 uM^-1.s^-1 | 10 s^-1 |  |
| CaM-Ca + Ca <===> CaM-Ca2 | 8.4846 uM^-1.s^-1 | 8.4853 s^-1 |  |
| CaM <===> CaM_xchange | 10 s^-1 | 10 s^-1 |  |
|  |  |  |  |
| Enzyme-reactions | Km | kcat | ratio |
| P_MAPK ---phosphatase--> MAPK | 0.02 uM | 1 s^-1 | 4 |
| APC ---P_MAPK--> AA | 5 uM | 10 s^-1 | 4 |
| K_A ---P_MAPK--> K_A_p | 10 uM | 10 s^-1 | 4 |
| inact_phosphatase ---P_MAPK--> reg_phosphatase | 1 uM | 0.1 s^-1 | 4 |
| MAPK ---act_PKC--> P_MAPK | 5 uM | 10 s^-1 | 4 |
| MAPK ---act_Raf--> P_MAPK | 20.001 uM | 10 s^-1 | 4 |
| P_MAPK ---reg_phosphatase--> MAPK | 0.099998 uM | 2 s^-1 | 4 |
|  |  |  |  |
| Pools |  |  |  |
| name | Initial Concen | buffered | D (μm^2/s) |
| phosphatase | 0.4 uM | No | 1 |
| P_MAPK | 0 uM | No | 1 |
| MAPK | 2 uM | No | 1 |
| AA | 0 uM | No | 1 |
| act_PKC | 0 uM | No | 0 |
| PKC | 1 uM | No | 1 |
| APC | 1 uM | Yes | 0 |
| K_A | 1 uM | No | 0 |
| Raf | 1.4 uM | No | 0 |
| act_Raf | 0 uM | No | 0 |
| Ca | 0.08 uM | No | 100 |
| Ca_input | 0.08 uM | Yes | 0 |
| K_A_p | 0 uM | No | 0 |
| inact_phosphatase | 1 uM | No | 1 |
| reg_phosphatase | 0 uM | No | 1 |
| CaM | 2 uM | No | 0.5 |
| CaM-Ca3 | 0 uM | No | 1 |
| CaM-Ca2 | 0 uM | No | 1 |
| CaM-Ca | 0 uM | No | 1 |
| CaM-Ca4 | 0 uM | No | 1 |
| CaM_xchange | 0 uM | No | 20 |
|  |  |  |  |

Electrical model: V in mV, referenced to resting potential. Time in ms.

Ion channel definitions, mostly from Traub, Wong, Miles, and Richardson. 1991. J. Neurophysiol 66:635-650.

gCa = gmaxCa.s^2^r ECa = 140

s-gate: $\alpha=\frac{1.6}{1+exp(-0.072\left( V-65 \right))}$ $\beta=\frac{0.02(V-51.1)}{\exp\left( \frac{V-51.1}{5} \right)-1}$

r-gate for $V\leq0$: $\alpha=0.005$ $\beta=0.0$

r-gate for $V>0$: $\alpha= \frac{exp(-\frac{V}{20})}{200}$ $\beta=0.005-\alpha$

gNa = gmaxNa.m^2^h ENa = 115

m-gate: $\alpha=\frac{0.32(13.1-V)}{\exp\left( \frac{13.1-V}{4} \right)-1}$ $\beta=\frac{0.28(V-40.1)}{\exp\left( \frac{V-40.1}{5} \right)-1}$

h-gate: $\alpha=0.128exp(\frac{17-V}{18})$ $\beta=\frac{4}{1+exp(\frac{40-V}{5})}$

gKDR = gmaxKDR.n EKDR = -15

n-gate: $\alpha=\frac{0.016(35.1-V)}{\exp\left( \frac{35.1-V}{5} \right)-1}$ $\beta=0.25exp(\frac{20-V}{40})$

gKAHP – gmaxKAHP.q EKAHP = -15

q-gate: $\alpha=min(20\times{10}^{-6}\left[ Ca \right], 0.01)$ $\beta=0.001$

gKA = gmaxKA.ab EKA = -15

a_gate: $\alpha=\frac{0.02(13.1-V)}{\exp\left( \frac{13.1-V}{10} \right)-1}$ $\beta=\frac{0.0175(V-40.1)}{\exp\left( \frac{V-40.1}{10} \right)-1}$

b-gate: $\alpha=0.0016exp(\frac{-13-V}{18})$ $\beta=\frac{0.05}{1+exp(\frac{10.1-V}{5})}$

gKC = gmaxKC.c EKC = -15

c_gate for V <= 50: $\alpha=\frac{exp(\frac{V-10}{11} - \frac{V-6.5}{27})}{18.975}$ $\beta=2exp(\frac{6.5-V}{27})$

c_gate for V > -50: $\alpha=2exp(\frac{6.5-V}{27})$ $\beta=0$

$g_{GluR}=\frac{A.{gmax}_{GluR}}{\tau1-\tau2} (exp({-t}/{\tau1)-exp({-t}/{\tau2}))}$ where

A = normalization constant such that gGluR = gmaxGluR at peak, and

τ1 = 2 τ2 = 9

$g_{GABAR}=\frac{A.{gmax}_{GABAR}}{\tau1-\tau2} (exp({-t}/{\tau1)-exp({-t}/{\tau2}))}$ where

A = normalization constant such that gGABAR = gmaxGABAR at peak, and

τ1 = 4 τ2 = 9

$g_{NMDAR}=\frac{{gmax}_{NMDAR}}{\tau} exp({-t}/{\tau)\frac{K_{Mg}}{K_{Mg}+[Mg]}}$ where

$K_{Mg}=\exp\left( (V-Erest)\gamma\right)/\eta$ τ = 20

γ = 0.28 η = 62

$I_{{NMDAR}_{Ca}}= g_{NMDAR}.\mathrm{Ca}_{\mathrm{frac}}.ln({[{Ca}_{out}]}/{[{Ca}_{in}]).V.\frac{\left[ {Ca}_{in} \right]-\varphi[{Ca}_{out}]}{(1-\varphi)(\left[ {Ca}_{in} \right]-[{Ca}_{out}])}}$ where

$\varphi=exp({-VFz}/{RT})$ F = 96485 sA/mol

z = 2 R = 8.314 J/(K.mol)

T = 300 Kelvin Ca_frac_= fraction of current carried at 0 mV by Ca = 0.02

[Ca_out] = 1.5mM [Ca_in] = 0.08 μM

Calcium pools:

d[Ca]/dt = φ(ICa + CaNMDA) –[Ca]/13.33

Passive properties

RM = 1.0 Ω.m^2^ RA = 1.0 Ω.m CM = 0.01 F/m^2^

Erest = -60 mV.

Channel distributions

| Channel | Zone | Distribution, i.e., Gmax. (S/m^2^) |
| --- | --- | --- |
| Ca | Basal + Apical | 0.0 Also tested 4.0 |
| Ca | Soma | 40 |
| Na | Basal | 60 |
| Na | Apical | 40+40exp(-p/200) |
| Na | Soma | 600 |
| K_DR | Basal | (p<400)*200 |
| K_DR | Apical | 60+40exp(p<125) |
| K_DR | Soma | 250 |
| K_AHP | All | 8 |
| K_C | Basal + Apical | 50+150exp(-p/200) |
| K_C | Soma | 100 |
| K_A | Basal + Apical | 50(1+2/(dia+0.1)) |
| K_A | Soma | 50 |
| GABA | All | 10+30(p < 125) |
| GluR | Spine Heads | 4000 |
| NMDAR | Spine Heads | 800 (Tested wide range) |

Where p = path length measured along dendrite, from soma to specified point on dendritic tree, and

dia = diameter of dendrite at specified point on dendritic tree.
