# Supplementary material for: Synaptic input sequence discrimination on behavioral timescales mediated by reaction-diffusion chemistry in dendrites
Source: eLife. 2017 Apr 19;6:e25827. doi: 10.7554/eLife.25827 (PMC5426902; doi:10.7554/eLife.25827)
Supplement: Source code 1. — The contents of the zip file include a README.txt, which has the file information and running instructions. In addition, it has script files for various parts of the figures, as well as model specification files for the chemical channel, and morphological parameters. Files include: DOI: http://dx.doi.org/10.7554/eLife.25827.019 [file elife-25827-code1.zip › Supplementary/SupplementaryMaterial.pdf]

Supplementary Material

Chemical model: Reduced MAPK system. All volumes are initial reference volumes, and are rescaled to actual volumes defined by detailed morphology of simulated neuron and subsequent spatial discretization for solving PDEs.

PSD	Vol = 0.01 fl		
Reactions	Kf	Kb	
Ca_input <===> Ca	500 s^-1	10 s^-1	
CaM-Ca3 + Ca <===> CaM-Ca4	1.8 uM^-1.s^-1	10 s^-1	
CaM + Ca <===> CaM-Ca	8.4846 uM^-1.s^-1	8.4853 s^-1	
CaM-Ca2 + Ca <===> CaM-Ca3	3.6001 uM^-1.s^-1	10 s^-1	
CaM-Ca + Ca <===> CaM-Ca2	8.4846 uM^-1.s^-1	8.4853 s^-1	
Pools			
name	Initial Concen	buffered	D (μm^2/s)
Ca	0.1 uM	No	100
Ca_input	0.08 uM	Yes	0
CaM	40 uM	No	0.5
CaM-Ca3	0 uM	No	1
CaM-Ca2	0 uM	No	1
CaM-Ca	0 uM	No	1
CaM-Ca4	0 uM	No	1
Spine Head	Vol = 0.09 fl		
Reactions	Kf	Kb	
CaM-Ca3 + Ca <===> CaM-Ca4	1.8 uM^-1.s^-1	10 s^-1	
CaM + Ca <===> CaM-Ca	8.4845 uM^-1.s^-1	8.4853 s^-1	
CaM-Ca2 + Ca <===> CaM-Ca3	3.6001 uM^-1.s^-1	10 s^-1	
CaM-Ca + Ca <===> CaM-Ca2	8.4845 uM^-1.s^-1	8.4853 s^-1	
CaM <===> CaM_xchange	1 s^-1	100 s^-1	
Pools			
name	InitialConc	buffered	D (μm^2/s)
Ca	0.11111 uM	No	100
CaM	40 uM	No	0.5
CaM-Ca3	0 uM	No	1
CaM-Ca2	0 uM	No	1
CaM-Ca	0 uM	No	1
CaM-Ca4	0 uM	No	1
CaM_xchange	0 uM	No	20
Dendrite	Vol = 1 fl		
Reactions	Kf	Kb	
AA <===> APC	0.4 s^-1	0.01 s^-1	
2 Ca + Raf <===> act_Raf	12 uM^-2.s^-1	4 s^-1	

K_A_p <===> K_A	0.05 s^-1	0 s^-1	
2 AA + PKC <===> act_PKC	1 uM^-2.s^-1	2 s^-1	
Ca_input <===> Ca	500 s^-1	10 s^-1	
reg_phosphatase <===> inact_phosphatase	0.03 s^-1	0 s^-1	
CaM-Ca3 + Ca <===> CaM-Ca4	1.8 uM^-1.s^-1	10 s^-1	
CaM + Ca <===> CaM-Ca	8.4846 uM^-1.s^-1	8.4853 s^-1	
CaM-Ca2 + Ca <===> CaM-Ca3	3.6 uM^-1.s^-1	10 s^-1	
CaM-Ca + Ca <===> CaM-Ca2	8.4846 uM^-1.s^-1	8.4853 s^-1	
CaM <===> CaM_xchange	10 s^-1	10 s^-1	
Enzyme-reactions	Km	kcat	ratio
P_MAPK ---phosphatase--> MAPK	0.02 uM	1 s^-1	4
APC ---P_MAPK--> AA	5 uM	10 s^-1	4
K_A ---P_MAPK--> K_A_p	10 uM	10 s^-1	4
inact_phosphatase ---P_MAPK--> reg_phosphatase	1 uM	0.1 s^-1	4
MAPK ---act_PKC--> P_MAPK	5 uM	10 s^-1	4
MAPK ---act_Raf--> P_MAPK	20.001 uM	10 s^-1	4
P_MAPK ---reg_phosphatase--> MAPK	0.099998 uM	2 s^-1	4
Pools			
name	Initial Concen	buffered	D (μm^2/s)
phosphatase	0.4 uM	No	1
P_MAPK	0 uM	No	1
MAPK	2 uM	No	1
AA	0 uM	No	1
act_PKC	0 uM	No	0
PKC	1 uM	No	1
APC	1 uM	Yes	0
K_A	1 uM	No	0
Raf	1.4 uM	No	0
act_Raf	0 uM	No	0
Ca	0.08 uM	No	100
Ca_input	0.08 uM	Yes	0
K_A_p	0 uM	No	0
inact_phosphatase	1 uM	No	1
reg_phosphatase	0 uM	No	1
CaM	2 uM	No	0.5
CaM-Ca3	0 uM	No	1
CaM-Ca2	0 uM	No	1
CaM-Ca	0 uM	No	1
CaM-Ca4	0 uM	No	1
CaM_xchange	0 uM	No	20

Electrical model: V in mV, referenced to resting potential. Time in ms.

Ion channel definitions, mostly from Traub, Wong, Miles, and Richardson. 1991. J. Neurophysiol 66:635-650.

gCa = gmaxCa.s^2r | ECa = 140

s-gate: $\alpha = \frac{1.6}{1+\exp(-0.072(V-65))}$ | $\beta = \frac{0.02(V-51.1)}{\exp\left(\frac{V-51.1}{5}\right)-1}$

r-gate for $V \leq 0$: $\alpha = 0.005$ | $\beta = 0.0$

r-gate for $V > 0$: $\alpha = \frac{\exp(-\frac{V}{20})}{200}$ | $\beta = 0.005 - \alpha$

gNa = gmaxNa.m^2h | ENa = 115

m-gate: $\alpha = \frac{0.32(13.1-V)}{\exp\left(\frac{13.1-V}{4}\right)-1}$ | $\beta = \frac{0.28(V-40.1)}{\exp\left(\frac{V-40.1}{5}\right)-1}$

h-gate: $\alpha = 0.128\exp(\frac{17-V}{18})$ | $\beta = \frac{4}{1+\exp(\frac{40-V}{5})}$

gKDR = gmaxKDR.n | EKDR = -15

n-gate: $\alpha = \frac{0.016(35.1-V)}{\exp\left(\frac{35.1-V}{5}\right)-1}$ | $\beta = 0.25\exp(\frac{20-V}{40})$

gKAHP – gmaxKAHP.q | EKAHP = -15

q-gate: $\alpha = \min(20 \times 10^{-6}[Ca], 0.01)$ | $\beta = 0.001$

gKA = gmaxKA.ab | EKA = -15

a_gate: $\alpha = \frac{0.02(13.1-V)}{\exp\left(\frac{13.1-V}{10}\right)-1}$ | $\beta = \frac{0.0175(V-40.1)}{\exp\left(\frac{V-40.1}{10}\right)-1}$

b-gate: $\alpha = 0.0016\exp(\frac{-13-V}{18})$ | $\beta = \frac{0.05}{1+\exp(\frac{10.1-V}{5})}$

gKC = gmaxKC.c | EKC = -15

c_gate for V <= 50: $\alpha = \frac{\exp(\frac{V-10}{11} - \frac{V-6.5}{27})}{18.975}$ $\beta = 2\exp(\frac{6.5-V}{27})$

c_gate for V > -50: $\alpha = 2\exp(\frac{6.5-V}{27})$ | $\beta = 0$

$g_{GluR} = \frac{A.gmax_{GluR}}{\tau1-\tau2}\ (\exp(-t/\tau1) - \exp(-t/\tau2))$ where
A = normalization constant such that gGluR = gmaxGluR at peak, and
τ1 = 2 | τ2 = 9

$g_{GABAR} = \frac{A.gmax_{GABAR}}{\tau1-\tau2}\ (\exp(-t/\tau1) - \exp(-t/\tau2))$ where
A = normalization constant such that gGABAR = gmaxGABAR at peak, and
τ1 = 4 | τ2 = 9

$g_{NMDAR} = \frac{gmax_{NMDAR}}{\tau} \exp(-t/\tau) \frac{K_{Mg}}{K_{Mg}+[Mg]}$ where

$K_{Mg} = \exp((V - Erest)\gamma) / \eta$

$\tau = 20$

$\gamma = 0.28$

$\eta = 62$

$I_{NMDAR_{Ca}} = g_{NMDAR}.\mathrm{Ca_{frac}}.\ln([Ca_{out}]/[Ca_{in}]).V.\frac{[Ca_{in}]-\varphi[Ca_{out}]}{(1-\varphi)([Ca_{in}]-[Ca_{out}])}$ where

$\varphi = \exp(-VFz/RT)$

F = 96485 sA/mol

z = 2

R = 8.314 J/(K.mol)

T = 300 Kelvin

Ca_{frac}= fraction of current carried at 0 mV by Ca = 0.02

[Ca_out] = 1.5mM

[Ca_in] = 0.08 μM

Calcium pools:

d[Ca]/dt = ϕ(ICa + CaNMDA) –[Ca]/13.33

Passive properties

RM = 1.0 Ω.m^2

RA = 1.0 Ω.m

CM = 0.01 F/m^2

Erest = -60 mV.

Channel distributions

Channel	Zone	Distribution, i.e., Gmax. (S/m^2)
Ca	Basal + Apical	0.0 Also tested 4.0
Ca	Soma	40
Na	Basal	60
Na	Apical	40+40exp(-p/200)
Na	Soma	600
K_DR	Basal	(p<400)*200
K_DR	Apical	60+40exp(p<125)
K_DR	Soma	250
K_AHP	All	8
K_C	Basal + Apical	50+150exp(-p/200)
K_C	Soma	100
K_A	Basal + Apical	50(1+2/(dia+0.1))
K_A	Soma	50
GABA	All	10+30(p < 125)
GluR	Spine Heads	4000
NMDAR	Spine Heads	800 (Tested wide range)

Where p = path length measured along dendrite, from soma to specified point on dendritic tree, and

dia = diameter of dendrite at specified point on dendritic tree.
